# Effect of swallowing related fatigue on eating and drinking behaviors across the age spectrum

**DOI:** 10.7717/peerj.20349

**Published:** 2026-01-30

**Authors:** Uzair Chilwan, K. Vijaya Kumar, Sudhin Karuppali, Venkataraja U. Aithal, Radish Kumar Balasubramanium

**Affiliations:** 1 Department of Audiology & Speech Language Pathology, Kasturba Medical College Mangalore, Manipal Academy of Higher Education, Manipal, India; 2 Department of Physiotherapy, Kasturba Medical College Mangalore, Manipal Academy of Higher Education, Manipal, India; 3 School of Rehabilitation and Medical Sciences, College of Health Sciences, University of Nizwa, Nizwa, Sultanate of Oman, Nizwa, Oman; 4 Department of Speech & Hearing, Manipal College of Health Professions, Manipal Academy of Higher Education, Manipal, India

**Keywords:** Swallowing, Fatigue, Older adults, Mealtime, Eating, Drinking, SERF

## Abstract

**Background:**

Swallowing related fatigue refers to the decline in swallowing efficiency and safety due to sustained muscular effort over time. It can significantly impact eating and drinking behaviors, potentially leading to aspiration, malnutrition and diminished quality of life. Understanding the effects of swallowing fatigue across the age spectrum can help guide clinical interventions and management strategies. Hence, this study aims to evaluate the effects of swallowing-related fatigue on eating and drinking behaviors in young, middle-aged and older adults.

**Methods:**

A cross-sectional study was conducted, recruiting 400 healthy individuals divided into three groups consisting of young adults, middle-aged adults and older adults. Participants with speech, language, swallowing, neurological, or cognitive impairments were excluded. The study utilized standardized assessments, including the Swallowing and Eating-Related Fatigue Scale (SERF) to measure swallowing fatigue. Objective swallowing function was evaluated using the Timed Water Swallow Test (TWST) for liquid intake, the Test of Masticating and Swallowing Solids (TOMASS) for solid food consumption, and the Mealtime Assessment Scale (MAS) to assess overall mealtime behaviors. Video recordings of swallowing tasks were analyzed to measure efficiency, speed, and fatigue-related changes.

**Results:**

Swallowing fatigue was significantly higher in older adults compared to middle-aged and young adults. TWST results showed that older adults exhibited longer swallowing durations, smaller bolus volumes per swallow, and reduced swallowing efficiency, though correlations between TWST parameters and fatigue were weak. In contrast, TOMASS scores revealed moderate associations with swallowing fatigue, as older adults took more bites, had longer mastication durations, and required more swallows per bolus. MAS scores demonstrated moderate to strong correlations with swallowing fatigue across all age groups, indicating that individuals experiencing higher fatigue levels displayed compromised mealtime efficiency and safety. Reliability analyses confirmed excellent test-retest reliability for TWST & TOMASS, with good to excellent interrater reliability.

**Conclusion:**

This study underscores the impact of swallowing-related fatigue on eating and drinking behaviors, particularly among older adults. While swallowing fatigue had minimal to moderate influence on TWST and TOMASS parameters respectively, MAS demonstrated stronger associations, suggesting that swallowing endurance plays a critical role in overall mealtime performance. These findings highlight the importance of integrating fatigue assessments into clinical dysphagia evaluations, as fatigue-related impairments may increase the risk of nutritional deficits and aspiration. Future research should focus on developing interventions to mitigate swallowing fatigue and improve mealtime efficiency, particularly in aging & clinical populations.

## Introduction

Swallowing is fundamental to meal consumption, encompassing the range of processes from oral preparation of food to its transport from the oral cavity, through the pharynx, past a closed larynx, and into the esophagus (Matsuo & Palmer, 2013). Swallowing continuously throughout a meal can be considered an endurance task, as it demands sustained attention along with the repetitive and continuous contraction of the diverse set of muscles dedicated to deglutition (Linda, 2014). Since swallowing and eating demand endurance, impaired endurance or easy fatigability can adversely affect these essential activities. Declines in swallowing performance can increase the risk of aspiration, while fatigue may necessitate premature cessation of eating, thereby elevating the risk of malnutrition and reducing the quality of life. Consequently, an individual’s ability to safely and efficiently consume a meal could be compromised. These significant health risks may be exacerbated in older adults and dysphagic populations where fatigue is already prevalent (Donini, Savina & Cannella, 2003).

Fatigue that manifests and/or is subjectively experienced during swallowing and eating is recognized as clinically significant in the field of dysphagia, or swallowing impairment. Lately, swallow fatigue has also been included in official practice guidelines as an important consideration during dysphagia evaluation (American Speech-Language-Hearing Association, 2025). A higher prevalence rate of 41.5% was observed in older adults in Indian population in a study recently conducted by Chilwan et al. (2025). Kluger, Krupp & Enoka (2013) had outlined the various factors that can contribute to the development of fatigue. This framework broadly characterizes two major domains, namely subjective experience of fatigue and performance fatigability (Goodpaster et al., 2006). Clinically, subjective experience deals with the perception of fatigue during swallowing. Clinicians may attribute any unexplained changes in performance during a meal or treatment session to “fatigue” or solely rely on patient reported measures such as Swallowing and Eating Related Fatigue scale (SERF) (Brates, Harel & Molfenter, 2022). Whereas performance fatiguability refers to the extent or speed of change in a performance metric compared to a baseline value during a specified period of task performance or mechanical output measurement (Goodpaster et al., 2006). This concept encompasses both physical and cognitive dimensions of fatiguability, both of which may be pertinent to executing swallowing function in elderly individuals. Effect of fatigue on swallowing performance can be objectively assessed using Quantitative measures of swallowing function such as Timed Water Swallow Test (TWST) and Test of Masticating and Swallowing Solids (TOMASS) which assesses the swallowing capabilities across liquids and solids respectively. These tests have already indicated age related changes in swallowing patterns across solids and liquids. It was observed that in TWST, after the age of 60, swallowing capacity and volume per swallow reduced, and time per swallow increased (Hughes & Wiles, 1996; Sarve, Krishnamurthy & Balasubramanium, 2021). On looking into the pattern across solid consistency, there was a significant age effect, with older adults taking more bites, performing more masticatory cycles, more swallows and needing more time to eat the cracker (Huckabee et al., 2018; Kothari et al., 2021). Both these tests were found to have higher test-retest reliability and inter-rater reliability.

Evaluation of natural conditions namely over the duration of meal using Mealtime Assessment Scale (MAS) (Pizzorni et al., 2020) will also provide greater insights about the swallow fatigue. It has multiple domains out of which it also targets fatigue as an important factor affecting the safety and efficacy of a swallow. The higher scores obtained in domain of swallowing safety and efficacy, the poorer the performance in swallowing skills across mealtime.

Despite the considerable importance given to swallowing related fatigue, there is limited evidence regarding its occurrence and effects on eating and drinking patterns. Based on findings from Chilwan et al. (2025), majority of older adults were found to agree that eating a full meal takes a lot of energy and also that fatigue affected their overall eating experience. Studies done by Brates, Harel & Molfenter (2022) and Chilwan et al. (2025) highlight an alarming prevalence rate of 75% and 41.5% respectively swallowing related fatigue among community dwelling older adults. Both these epidemiological related studies however did not assess the impact of swallowing fatigue on eating and drinking behaviours. Consequently, clinical methods for identifying, improving or mitigating the negative effects of fatigue during mealtimes lack sufficient support. Hence the current study aims to evaluate and assess the impact of swallowing related fatigue on eating and drinking pattern across healthy young, middle-aged and older adults.

## Materials & Methods

### Study design

The current study utilized a cross-sectional design with a non-randomized purposive sampling procedure. The study was conducted between June 2024 and January 2025. The study received approval from institutional ethics committee of Kasturba Medical College Mangalore (IECKMCMLR-11/2022/452).

### Participants

A total of 400 participants were included, and categorized into three groups: 127 healthy young adults ranging from 18–40 years (Group A), 124 healthy middle-aged adults, ranging from 41–59 years (Group B) and 149 healthy older adults 60 years and above (Group C). Participants were recruited as healthy volunteers from the community. Specifically, older adults were approached from local old age homes and yoga centers, while the young and middle-aged adults were recruited from general community. Written informed consent was obtained from the participants prior to collecting relevant data. Participants with any speech, language swallowing or cognitive deficits as well as those who avoid hard food textures and have gluten intolerance were excluded from the study. All participants underwent neurological screening that included detailed case history and structured cranial nerve examination (cranial nerve functions, motor and sensory status) performed by the principial investigator (a Speech-Language Pathologist). This process ensured the exclusion of individuals with neurological conditions. Participants who are on non-oral modes of feeding, or have any surgery to the oral structures involved in swallowing were excluded from the study as well.

### Materials

The TWST utilized 150 ml of room temperature bottled mineral water which was provided in a plastic cup. For the TOMASS test, a Parle Monaco cracker, measuring 4.4 cm in diameter and weighing 6.67 g was used. Parle Monaco TM is a commercially available cracker commonly used in India for the reliable assessment of mastication and swallowing in adults. The timing was recorded using a stopwatch.

### Procedure

The participants were provided the SERF scale to evaluate the severity of swallowing related fatigue. After filling the form, the study cohort was then subjected to the following quantitative tests, *i.e.,* TWST & TOMASS.

• Participants were seated comfortably during the assessments. For assessing the liquid consistency, the participants were provided with 150 ml of water in a paper cup with the instruction to drink “as quickly and comfortably as possible”. Temporal measures were recorded from the moment the cup touched the patient lips till the last laryngeal elevation post swallow. Number of swallows were documented by visual observation of thyroid cartilage displacement.
• To assess the masticatory efficiency for solids, we provided the participants with one cracker with the instruction, ‘consume this cracker as quickly and comfortably and when you are done with swallowing the cracker, say out your name loud and clear’. From this test we measured the number of bites, number of masticatory cycles taken for that cracker, number of swallows and most importantly the amount of time taken to consume the cracker.

Both the procedures were video recorded for analysis purposes. A digital camera (AUSHA^®^ 5K sport action camera) was mounted on a tripod (Digitek DTR 520BH) to capture four quadrants involving the head and neck. The researcher adjusted the frame of capture until the following landmarks were present in the frame, the head superiorly, clavicle inferiorly, nose anteriorly, and ear posteriorly.

• Lastly to assess the swallowing safety and efficacy of swallowing, the mealtime assessment scale (MAS) was administered by the researcher. Here, the participants were asked to complete a normal meal, whereas the researcher observed the behaviors and the patterns of eating and completing a meal by the participant. The meal comprised of a typical staple diet covering rice or medium sized chapati, lentils, and vegetables ranging up to (∼120 calories per food item).

For interrater reliability, 20% (*n* = 78) of the samples were chosen randomly, and analyzed by a second rater who was a post graduate student blinded to the analysis conducted by the first rater. The measures that were included for the reliability studies (interrater reliability and test–retest reliability were the parameters of TWST and TOMASS.

SERF has been formulated to evaluate swallowing related fatigue wherein the first six questions target the localized fatigue in swallowing musculature and last six questions target the general fatigue in terms of mealtime and behavior (Brates, Harel & Molfenter, 2022). The list of questions of SERF has been listed out in Table 1. Hence, we decided to analyze the objective tests by correlating it specifically with localized fatigue as well as fatigue related to mealtime behaviors. Spearman Correlation was carried out to assess the degree and strength of correlation between the quantitative tests and severity of swallowing related fatigue.

## Results

The aim of the study was to determine the impact of swallowing related fatigue and its effect on eating and drinking behaviors across three groups namely, young healthy adults, healthy middle-aged adults and healthy older adults. Descriptive statistics was carried out for all the continuous variables covered in the study. Shapiro Wilks test was done to check for normality among the variables. All the variables had *p* < 0.05 indicating the data is not normally distributed. Spearmann correlation coefficient was carried out to check for relationship of localized swallowing fatigue and general swallowing fatigue across the three quantitative tests. Linear regression was carried out to assess the effect of age, gender and swallowing fatigue on the drinking, eating and mealtime.

**Table 1:** Swallowing and eating related fatigue scale.

Localized fatigue	Chewing makes my jaw feel tired.
	There are foods or drinks I avoid because they take too much effort to swallow.
	My throat feels tired by the end of a meal.
	Swallowing feels more effortful at the end of a meal.
	If I eat when feeling tired/fatigued, I cough more than usual.
	If I eat when feeling tired/fatigued, food sticks in my throat more than usual.
General fatigue	I stop eating meals because I feel tired, even if I am not finished.
	I take breaks during meals because I feel tired.
	I skip meals if I’m feeling too tired/fatigued.
	I find that I am able to eat more when I feel well-rested.
	Eating a full meal takes a lot of energy.
	Fatigue impacts my overall eating experience.

### Gender wise distribution and prevalence of swallowing related fatigue

Group A included 127 healthy young adults (62 males, 65 females). Group B had 124 healthy middle-aged adults (49 males, 75 females) and Group C was comprised of 149 healthy older adults (68 males, 81 females).

Based on the findings from the SERF scores, we observed the following prevalence rates across the three groups with healthy young adults reporting a prevalence rate of 15.74%. Middle-aged adults and older adults were found to have prevalence rates of 24.1% and 40.26%, respectively.

### Descriptive statistics

Descriptive statistics for all the variables from the quantitative tests have been summarized in Table 2.

**Table 2:** Descriptive statistics for continuous variables across different Timed Water Swallow Test, Test of Mastication and Swallowing in Solids and Mealtime Assessment Scale.

**Parameter**	**Groups**	***N***	**Mean**	**Median**	**SD**	**Min**	**Max**	**Shapiro Wilk (p)**
**Timed Water Swallow Test**								
Volume per swallow (ml/n)	Group A	127	27.7	25	11.8	6.81	75	<0.001
	Group B	124	28.6	25	10.5	8.33	50	<0.001
	Group C	149	26.8	25	13.2	7.5	75	<0.001
Time per swallow (sec/n)	Group A	127	1.84	1.65	0.69	0.91	4.6	<0.001
	Group B	124	2.1	1.94	0.82	1.06	5.72	<0.001
	Group C	149	2.64	2.19	1.53	1.06	12.1	<0.001
Swallow capacity (ml/sec)	Group A	127	16.3	15.7	6.35	4.65	38.2	0.003
	Group B	124	14.9	14.3	5.97	2.35	27.8	0.004
	Group C	149	11.6	11	5.36	1.51	28.1	<0.001
**Test of Mastication and Swallowing in Solids (TOMASS)**								
Discrete bites per cracker (n)	Group A	127	1.74	2	0.89	1	6	<0.001
	Group B	124	2.1	1.5	1.46	1	6	<0.001
	Group C	149	2.52	2	1.7	1	8	<0.001
Masticatory cycles per cracker (n)	Group A	127	28.4	26	11.1	13	70	<0.001
	Group B	124	33.2	30.5	11.5	13	72	<0.001
	Group C	149	50	46	20.9	16	153	<0.001
Swallows per cracker (n)	Group A	127	2.45	2	1.51	1	7	<0.001
	Group B	124	2.57	2	1.44	1	7	<0.001
	Group C	149	2.88	2	1.61	1	9	<0.001
Total time (sec)	Group A	127	29.3	26.9	10.9	12.7	75.4	<0.001
	Group B	124	33.6	29.6	13.3	14.5	73.6	<0.001
	Group C	149	48.6	45	20.6	17	165	<0.001
**Mealtime Assessment Scale**								
Safety score (%)	Group A	83	1.00	0	3.02	0	16.7	<0.001
	Group B	67	3.36	0	6.81	0	25	<0.001
	Group C	54	20.1	20.8	16.3	0	66.7	0.001
Efficacy score (%)	Group A	83	1.20	0	2.88	0	11.1	<0.001
	Group B	67	3.98	0	7.35	0	44.4	<0.001
	Group C	54	24.6	25	16.9	0	61.1	0.009
Meal duration (mins)	Group A	83	15.1	15	3.11	10	250	<0.001
	Group B	67	18.8	20	3.28	12	29	<0.001
	Group C	54	23	23.5	6.13	12	40	0.003

From Table 2 we can summarize the older adults had significantly poorer values in terms of swallow safety, efficacy and mealtime duration.

### Inter rater and test-retest reliability

Table 3 depicts a summary of ICC values for all the parameters on TWST and TOMASS. As shown, the ICC values ranged from 0.71–0.98 across various parameters hereby indicating a moderate to high level of correlation for analysis amongst the two reviewers.

**Table 3:** Test-retest and inter-rater reliability values of Timed Water Swallow Test (TWST) and Test of Mastication and Swallowing in Solids (TOMASS).

**Parameter**	**Cronbach’s alpha**	**Intra class correlation**
**Timed water swallow test**		
Volume per swallow	0.859	0.753
Time per swallow	0.834	0.715
Swallow capacity	0.979	0.958
**Test of mastication and swallowing in solids**		
Number of bites	0.976	0.953
Number of masticatory cycles	0.969	0.94
Number of swallows	0.824	0.71
Total time	0.99	0.98

Table 3 shows very good to excellent test-retest reliability across both the quantitative tests. However, in terms of inter-rater reliability a wide variation was seen ranging from good to excellent inter-rater reliability.

### Correlation analysis between swallowing related fatigue on drinking, eating and mealtime

**Figure 1:**
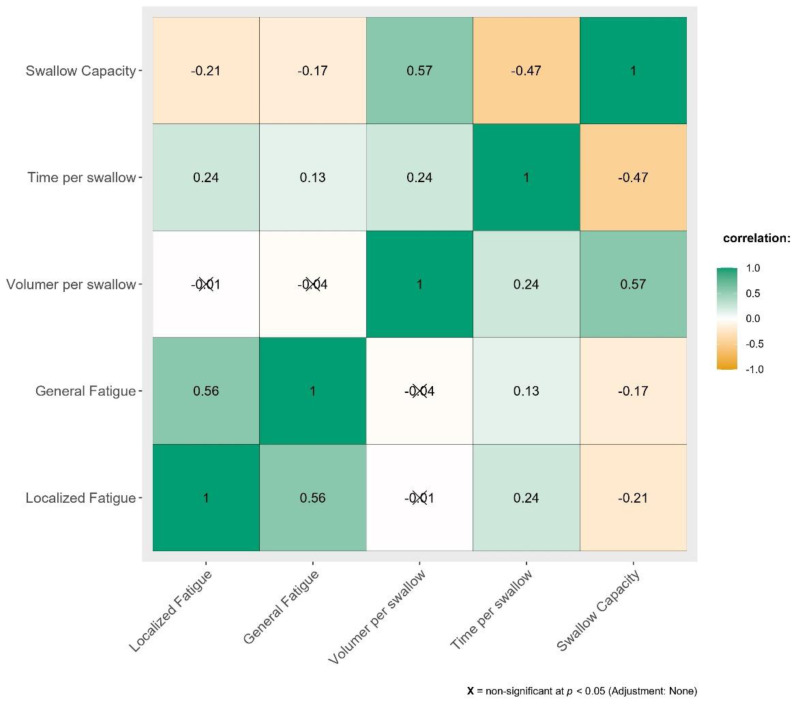
Correlation between SERF scores and parameters of Timed Water Swallowing Test (TWST).

Correlation between severity of swallowing related fatigue and its effect on drinking has been summarized in Fig. 1.

Figure 1 depicts very weak to weak correlation for TWST parameters against localized and general swallowing fatigue.

**Figure 2:**
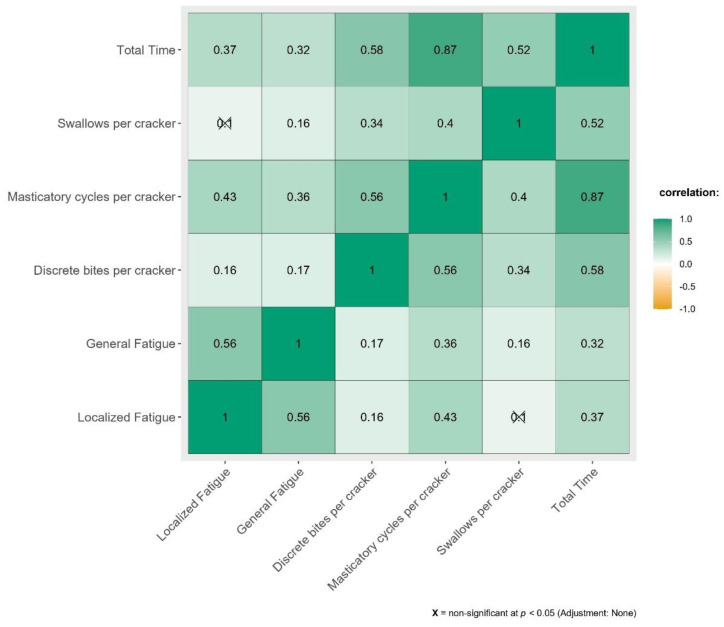
Correlation between SERF scores and parameters of Test of Masticating and Swallowing in Solids (TOMASS).

The effect of swallowing related fatigue on eating pattern across all parameters of TOMASS have been depicted in Fig. 2.

Figure 2 demonstrates weak to moderate correlation between TOMASS parameters against swallowing fatigue.

**Figure 3:**
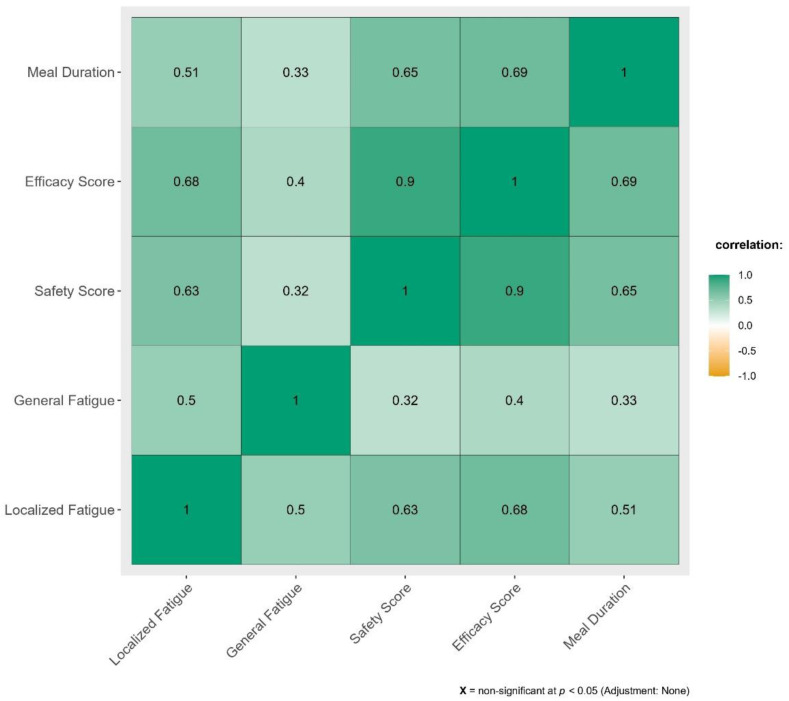
Correlation between SERF scores and Mealtime Assessment Scale (MAS).

Impact of swallowing related fatigue across mealtime has been illustrated in Fig. 3.

From Fig. 3 we can determine that moderate to strong positive correlation exists between the domains in MAS against swallowing related fatigue.

### Effect of age, gender and swallowing fatigue on drinking, eating and mealtime

A linear regression analysis was conducted to examine the effect of gender, age, and swallowing fatigue on drinking-related swallowing parameters: volume per swallow, time per swallow, and swallow capacity. The results have been summarized in Table 4.

**Table 4:** Effect of age, gender and swallowing fatigue on drinking task.

			**Confidence interval**		
**Predictor**	**Estimate**	**SE**	**Lower**	**Upper**	***p***
**Volume per swallow**					
Intercept	27.103	1.687	23.785	30.422	<0.001
SERF	−0.046	0.111	−0.265	0.176	0.673
Age	−0.016	0.034	−0.083	0.0502	0.625
Gender					
Male-Female	3.9784	1.19	1.638	6.318	<0.001
**Time per swallow**					
Intercept	1.2057	0.156	0.898	1.513	<0.001
SERF	0.0124	0.0102	−0.007	0.032	0.231
Age	0.0168	0.003	0.0106	0.023	<0.001
Gender					
Male-Female	0.1515	0.110	−0.0652	0.3681	0.170
**Swallow capacity**					
Intercept	18.637	0.828	17.008	20.267	<0.001
SERF	−0.072	0.0546	−0.180	0.0351	0.187
Age	−0.092	0.0167	−0.125	−0.0596	<0.001
Gender					
Male-Female	1.57	0.5845	0.421	2.7196	0.005

From Table 4, the overall model revealed that gender significantly influenced both volume per swallow and swallow capacity, with males performing better in these domains. Age was significantly associated with increased time per swallow and decreased swallow capacity. In contrast, swallowing fatigue did not show a statistically significant effect on any of the drinking-related swallowing measures.

Similarly, a linear regression analysis was conducted to examine the influence of gender, age, and swallowing fatigue on various eating task parameters, including discrete bites, masticatory cycles, swallows per cracker, and total time which have been listed out in Table 5.

**Table 5:** Effect of age, gender and swallowing fatigue on eating task.

			**Confidence interval**		
**Predictor**	**Estimate**	**SE**	**Lower**	**Upper**	***p***
**Discrete bites per cracker**					
Intercept	1.531	0.1968	1.1445	1.9183	<0.001
SERF	0.018	0.0129	−0.0071	0.0437	0.159
Age	0.014	0.0039	0.0065	0.0221	<0.001
Gender					
Male-Female	−0.577	0.1388	−0.8504	−0.3045	<0.001
**Masticatory cycles per cracker**					
Intercept	14.324	2.1085	10.179	18.469	<0.001
SERF	0.683	0.1388	0.410	0.956	<0.001
Age	0.396	0.0425	0.313	0.480	<0.001
Gender					
Male-Female	−4.376	1.4876	−7.301	−1.452	0.003
**Swallows per cracker**					
Intercept	2.4422	0.2144	2.0227	2.8657	<0.001
SERF	0.0363	0.0141	0.0086	0.0641	0.010
Age	0.002	0.0432	−0.0064	0.0105	0.643
Gender					
Male-Female	−0.4523	0.1512	−0.7497	−0.155	0.003
**Total time**					
Intercept	19.203	2.1318	15.012	23.394	<0.001
SERF	0.552	0.1403	0.276	0.828	<0.001
Age	0.353	0.0429	0.268	0.437	<0.001
Gender					
Male-Female	−8.238	1.5041	−11.195	−5.281	<0.001

Gender, age, and swallowing fatigue significantly impacted multiple components of the eating task. Males demonstrated greater oral efficiency, with fewer bites, chewing cycles, swallows, and shorter total eating time. Age was associated with increased oral effort and duration. Notably, swallowing fatigue showed significant effects on masticatory effort, number of swallows, and eating duration, indicating that perceived fatigue can influence real-time eating behaviors.

Finally, linear regression analysis was performed to evaluate the influence of gender, age, and swallowing fatigue on mealtime outcomes namely swallowing safety, swallowing efficacy, and total mealtime duration, which has been illustrated in Table 6.

**Table 6:** Effect of age, gender and swallowing fatigue on mealtime.

			**Confidence interval**		
**Predictor**	**Estimate**	**SE**	**Lower**	**Upper**	***p***
**Safety score**					
Intercept	−1.4291	0.2017	−1.8269	−1.0313	<0.001
SERF	0.076	0.015	0.0464	0.1056	<0.001
Age	0.0351	0.0045	0.0262	0.0441	<0.001
Gender					
Male-Female	0.0669	0.1559	−0.2405	0.3744	0.668
**Efficacy score**					
Intercept	−2.5278	0.3089	−3.137	−1.9186	<0.001
SERF	0.1578	0.0229	0.1125	0.2031	<0.001
Age	0.0602	0.0069	0.0465	0.0739	<0.001
Gender					
Male-Female	−0.0570	0.2387	−0.5278	0.4137	0.812
**Mealtime duration**					
Intercept	4.944	0.689	3.5852	6.302	<0.001
SERF	0.158	0.0512	0.0565	0.259	0.002
Age	0.156	0.0155	0.1256	0.187	<0.001
Gender					
Male-Female	0.142	0.5324	−0.9084	1.191	0.791

From Table 6 it was seen that in contrast to the drinking and eating tasks, gender did not significantly influence any of the mealtime outcomes. Swallowing fatigue and increasing age emerged as consistent and significant predictors of compromised mealtime performance, manifesting as reduced safety and efficacy as well as prolonged meal duration.

### Comparative analysis of drinking, eating, and mealtime performance across age groups

Kruskal Wallis test was carried out to check for inter group differences on the quantitative tests across the age groups. The results have been summarized along with *post hoc* results in the Tables 7 and 8.

**Table 7:** Group comparisons of drinking, eating, and mealtime metrics using Kruskal–Wallis test.

**Parameter**	*χ* ^2^	***p***
**Timed water swallow test**		
Volume per swallow	4.73	0.094
Time per swallow	42.54	<0.001
Swallow capacity	45.42	<0.001
**Test of mastication and swallowing in solids**		
Discrete bites per cracker	13.43	0.001
Masticatory cycles per cracker	116.83	<0.001
Swallows per cracker	7.07	0.029
Total time	99.83	<0.001
**Mealtime assessment scale**		
Safety score	89.73	<0.001
Efficacy score	101.14	<0.001
Mealtime duration	73.30	<0.001

**Table 8:** Pairwise comparisons across groups using the Dwass-Steel-Critchlow-Flinger test for drinking, eating, and mealtime parameters.

			**W**	***p***
**Timed Water Swallow Test**				
Pairwise comparison volume per swallow				
GROUP A		GROUP B	1.63	0.481
GROUP A		GROUP C	−1.52	0.530
GROUP B		GROUP C	−3.01	0.084
Pairwise comparison Time per swallow				
GROUP A		GROUP B	4.17	0.009
GROUP A		GROUP C	9.08	<0.001
GROUP B		GROUP C	5.03	0.001
Pairwise comparison swallow capacity				
GROUP A		GROUP B	−2.28	0.241
GROUP A		GROUP C	−9.10	<0.001
GROUP B		GROUP C	−6.67	<0.001
**Test of Mastication and Swallowing in Solids**				
Pairwise comparison discrete bites per cracker				
GROUP A		GROUP B	1.42	0.577
GROUP A		GROUP C	5.18	<0.001
GROUP B		GROUP C	3.17	0.064
Pairwise comparison masticatory cycles per cracker				
GROUP A		GROUP B	5.25	<0.001
GROUP A		GROUP C	14.07	<0.001
GROUP B		GROUP C	10.87	<0.001
Pairwise comparison swallows per cracker				
GROUP A		GROUP B	1.45	0.561
GROUP A		GROUP C	3.69	0.025
GROUP B		GROUP C	2.23	0.255
Pairwise comparison total time				
GROUP A		GROUP B	3.76	0.021
GROUP A		GROUP C	13.30	<0.001
GROUP B		GROUP C	10.03	<0.001
**Mealtime Assessment Scale**				
Pairwise comparison safety score				
GROUP A		GROUP B	3.45	0.039
GROUP A		GROUP C	12.24	<0.001
GROUP B		GROUP C	9.54	<0.001
Pairwise comparison efficacy score				
GROUP A		GROUP B	4.44	0.005
GROUP A		GROUP C	13.11	<0.001
GROUP B		GROUP C	10.37	<0.001
Pairwise comparison mealtime duration				
GROUP A		GROUP B	8.56	<0.001
GROUP A		GROUP C	10.55	<0.001
GROUP B		GROUP C	6.01	<0.001

The Kruskal-Wallis test revealed significant group differences in several parameters related to swallowing, eating, and mealtime performance. Within the Timed Water Swallow Test, there were statistically significant differences between groups for time per swallow (*χ*^2^ = 42.54, *p* < 0.001) and swallow capacity (*χ*^2^ = 45.42, *p* < 0.001), while volume per swallow did not differ significantly (*χ*^2^ = 4.73, *p* = 0.094). *Post-hoc* pairwise comparisons using the Dwass-Steel-Critchlow-Flinger test showed that time per swallow progressively increased across all three groups: Group A *vs* B (*p* = 0.009), A *vs* C (*p* < 0.001), and B *vs* C (*p* = 0.001), suggesting a gradual decline in speed of swallowing with group progression, likely due to age. Similarly, swallow capacity was significantly reduced in Group C when compared with Group A (*p* < 0.001) and Group B (*p* < 0.001), indicating impaired efficiency in older adults.

In the TOMASS, group differences were evident across all the parameters. However, *post-hoc* analysis revealed that Group C required significantly more bites than Group A (*p* < 0.001). Masticatory cycles increased significantly between all pairs (A *vs* B, A *vs* C, B *vs* C; all *p* < 0.001), suggesting that chewing effort increases progressively across groups. Swallows per cracker were significantly higher in Group C compared to Group A (*p* = 0.025), and total time to complete a cracker was significantly longer for each group comparison: A *vs* B (*p* = 0.021), A *vs* C (*p* < 0.001), and B *vs* C (*p* < 0.001).

The MAS also revealed significant group differences in swallowing safety (*χ*^2^ = 89.73, *p* < 0.001), efficacy (*χ*^2^ = 101.14, *p* < 0.001), and mealtime duration (*χ*^2^ = 73.30, *p* < 0.001). All three parameters showed significant pairwise differences across all group comparisons. For safety scores, Group B had higher scores than Group A (*p* = 0.039), Group C had significantly higher scores than both A (*p* < 0.001) and B (*p* < 0.001), indicating progressive decline in swallowing safety. Efficacy scores were also significantly worse in Group B compared to Group A (*p* = 0.005), and further worsened in Group C compared to both A and B (*p* < 0.001). Likewise, mealtime duration increased significantly between all groups, with Group C taking the longest time to complete meals (A *vs* B, A *vs* C, B *vs* C; all *p* < 0.001).

In summary, the results demonstrate that swallowing, chewing, and mealtime performance significantly decline across groups, with Group C (older adults) showing the most marked impairments. These include slower and less efficient water swallowing, increased oral effort during solid bolus management, greater swallowing safety and efficacy concerns, and prolonged mealtime durations. Group B (middle-aged adults) often exhibited intermediate performance between the younger and older groups, reflecting a possible transition in functional swallowing capacity with aging.

## Discussion

The aim of the current study was to assess the impact of swallowing fatigue on eating and drinking patterns among healthy young, middle aged and older adults. The hypothesis was that older adults will have poorer swallowing performance on TWST, TOMASS and MAS due to increased perception of swallowing fatigue as compared to healthy middle-aged adults and healthy young adults.

The findings of this study add on to the existing literature that older adults have reported higher prevalence rates for swallowing related fatigue (Brates, Harel & Molfenter, 2022; Chilwan et al., 2025).

Very weak to weak correlation was found between parameters of Timed Water Swallow Test and swallowing related fatigue. TWST focuses on swallowing water over a short duration, which might not replicate real-world conditions of eating or drinking over extended periods, where swallowing fatigue could become more evident. Swallowing fatigue may manifest in prolonged or repetitive swallowing tasks, not necessarily in a short-duration TWST (Leder et al., 2012).

Furthermore, individuals, mainly older adults experiencing fatigue might unconsciously adjust their swallowing rate or style during TWST to maintain efficiency, which could mask the correlation between fatigue and TWST parameters (Sella Weiss, Gvion & Mcrae, 2021).

The TOMASS involves a single type of food, cracker, which may not also fully represent the complexity of swallowing fatigue experienced across varied foods and consistencies. Discrete bites and swallows are coarse-grained measures and may not capture subtle changes in mastication or swallowing efficiency caused by fatigue. Biting and swallowing a cracker might demand less effort and be less fatiguing compared to repetitive or prolonged meal tasks, limiting the correlation with perceived swallowing fatigue. Individuals experiencing swallowing-related fatigue may adapt compensatory strategies in their biting or swallowing behaviors by taking smaller bites and longer pauses to maintain task efficiency hence, masking a direct correlation (Solomon, 2006). Weak correlation was hence obtained on parameters namely, number of swallows and discrete bites per cracker and swallowing related fatigue.

Although TOMASS parameters such as discrete bites, no of swallows did not correlate with SERF scores, there were two significant relationships found between the TOMASS measurements and the swallowing related fatigue scores that are worth exploring. Moderate correlation was observed on TOMASS parameters namely, total time and number of masticatory cycles. Increased swallowing fatigue could directly affect how long it takes to chew and swallow a solid bolus, as fatigue may slow down or disrupt typical motor patterns. Fatigue might lead to less efficient chewing, requiring more cycles to achieve a swallowable bolus, thus reflecting a moderate link with fatigue. Studies have shown that muscle fatigue diminishes bite force and efficiency, particularly in tasks requiring repetitive chewing or clenching (Farella et al., 2010). Fatigue can reduce the efficiency of masticatory and swallowing muscles, resulting in prolonged chewing or swallowing efforts, leading to more cycles or increased time. This can be due to a decline in total muscle cross-sectional area, with reductions of approximately 40% observed between the ages of 20 and 80 years (Roos, Rice & Vandervoort, 1997). This decrease is primarily attributed to the loss of functional motor units (Campbell, McComas & Petito, 1973). These parameters, total time and masticatory cycles inherently reflect aspects of muscular endurance, which overlaps with the concept of swallowing-related fatigue.

Swallowing-related fatigue should be considered within the broader context of the mealtime experience, as it is influenced by the interplay of homeostatic factors, such as hunger, and psychological factors, including mood and motivation, which collectively contribute to the perception of fatigue during swallowing (Brates, Troche & Molfenter, 2020). Unlike isolated tests such as TWST or TOMASS, the Mealtime Assessment Scale evaluated performance in realistic eating scenarios. Effects of swallowing fatigue, which accumulate over a meal, are more pronounced and directly measurable in the domains covered in MAS.

The first six items of the SERF scale, focusing on localized fatigue, specifically captured perceptions of swallowing fatigue in the oral musculature, including the jaw, cheeks, and pharynx. Symptoms such as jaw tiredness, throat fatigue, food sticking post-meal, and coughing due to fatigue were significantly associated with the MAS domains of swallow safety and swallow efficacy. A strong positive correlation suggests that localized fatigue directly impacts the functional ability to control the bolus, manage residue, and maintain safety during swallowing. This finding aligns with previous research indicating that oral fatigue contributes to compromised airway protection and increased aspiration risk (Ibrahim et al., 2018).

The second set of SERF scale items addressed generalized fatigue, capturing behaviors such as skipping meals, taking breaks, and the impact of fatigue on overall eating experience. These items demonstrated a moderate correlation with MAS findings, particularly in the swallow efficacy domain. While generalized fatigue affects the ability to sustain mealtime activities, its influence on specific swallowing safety parameters appears less pronounced. These results are consistent with studies emphasizing the role of systemic fatigue in altering eating habits and mealtime duration rather than directly impairing swallowing safety mechanisms (Brates & Molfenter, 2021).

Thus, Mealtime duration serves as a vital metric in understanding the efficiency and challenges associated with swallowing and eating, particularly in individuals experiencing swallowing related fatigue. This study observed a significant relationship between mealtime duration and fatigue, with extended durations correlating with higher scores on the SERF scale. These findings underscore the multifactorial nature of prolonged mealtime durations (Brates, Troche & Molfenter, 2020). Prolonged mealtime durations are often indicative of the need for additional time to chew, prepare, and swallow food safely and effectively. During the oral phase of ingestion, mastication, tongue mobility, and lip closure play critical roles in bolus formation. However, these functions tend to decline with advancing age (Fucile et al., 1998).

In this study, individuals reporting higher levels of generalized fatigue on the SERF scale were more likely to take breaks during meals and required more time to complete them. This pattern reflects the physical and systemic toll of fatigue on swallowing efficiency (Crow & Ship, 1996; Hara et al., 2018). Previous research has also shown that generalized fatigue reduces the stamina needed to sustain the complex motor tasks of eating, thereby increasing the overall time required for meal completion (Hara et al., 2018).

### Limitations and future directions

This study has certain limitations that should be acknowledged. First, instrumental assessments of the pharyngeal phase, such as VFSS or FEES, were not used, which limits insight into deeper swallowing dynamics. Second, BMI was not collected, which could have provided further understanding of fatigue contributors. Additionally, denture status was not documented, despite its relevance to mastication efficiency in older adults. Future studies should aim to include these variables to enhance clinical interpretation and applicability.

The task-specific nature of the Timed Water Swallow Test and the Test of Mastication and Swallowing of Solids may have restricted the ability to fully capture the cumulative and complex effects of swallowing fatigue experienced during prolonged or varied mealtime tasks. Additionally, both tests primarily assess oral and preparatory phases of swallowing, potentially overlooking deficits in the pharyngeal and esophageal phases where fatigue might be more pronounced. The inclusion of a heterogeneous population comprising healthy young, middle-aged, and older adults may have diluted the observed correlations due to age-related differences in muscle strength, endurance, and baseline fatigue levels. Psychological factors such as motivation, mood, and stress, as well as environmental influences like the mealtime setting, were not controlled for, which could have impacted perceived fatigue and swallowing performance. Future research should explore longitudinal effects of fatigue management interventions on SERF and MAS scores and investigate their predictive value in clinical populations.

## Conclusions

This study highlights the impact of swallowing-related fatigue on eating and drinking performance across age groups, with localized fatigue showing a strong correlation with the MAS domains of swallow safety and efficacy, and generalized fatigue demonstrating moderate correlations with mealtime behaviors. The weak correlations observed with TWST and TOMASS suggest that these tools may not fully capture fatigue effects in real-world contexts. These findings underscore the importance of addressing swallowing fatigue through targeted interventions, such as oral motor exercises and meal pacing strategies, to optimize swallowing safety and efficiency. Incorporating fatigue assessments and assessing them with MAS in outpatient and bedside settings can help identify patients who may benefit from interventions aimed at improving stamina, meal pacing, and safe intake.

##  Supplemental Information

10.7717/peerj.20349/supp-1 Supplemental Information 1 Data

## Additional Information and Declarations

### Data Availability

The following information was supplied regarding data availability: The raw data is available in the Supplemental File.
